# Emerging Evidence Suggests That N-Glycosylation Pathway Perturbation Has a Greater Impact than Microbial Depletion on α-Gal Antigen Biogenesis in Lone Star Tick (*Amblyomma americanum*) Salivary Glands

**DOI:** 10.3390/microorganisms14102375

**Published:** 2026-10-16

**Authors:** Surendra Raj Sharma, Shahid Karim

**Affiliations:** 1 School of Biological, Environmental and Earth Sciences, The University of Southern Mississippi, Hattiesburg, MS 39406, USA; surendra.sharma@med.unc.edu; 2 Center for Molecular Medicine, University of North Carolina at Chapel Hill, Chapel Hill, NC 27599, USA

**Keywords:** ticks, *Amblyomma americanum*, microbial dysbiosis, glycosylation, α-gal syndrome, alpha-gal antigens, hypersensitivity

## Abstract

Alpha-gal syndrome (AGS) is an emerging tick-associated allergy characterized by IgE-mediated hypersensitivity to galactose-α-1,3-galactose (α-gal). Although α-gal antigens have been identified in *Amblyomma americanum* salivary glands, the mechanisms governing their production during feeding remain unresolved. Here, we investigated whether the tick core microbes or endogenous tick glycosylation pathways contribute to α-gal antigen biogenesis. Microbial depletion was induced by feeding larvae and nymphs on tetracycline-treated α-gal-deficient mice, followed by molting into adults; adult ticks were subsequently injected with tetracycline and maintained under microbiota-reduced conditions. In parallel, inhibition of N-linked glycosylation and galactosyltransferase activity using tunicamycin and 2-deoxy-2-fluoro-D-galactose (dGalF), respectively, was used to assess glycosylation-dependent mechanisms. α-gal-associated antigens were further evaluated by immunoblotting and confocal microscopy using an anti-α-gal antibody. Despite a marked reduction in bacterial burden in immature and adult ticks, tetracycline treatment α-gal-associated antigen remained detectable in partially fed nymph and adult salivary glands. Treatment with tunicamycin or dGalF reduced α-gal-associated antigen levels, as determined by Western blot analysis, whereas microbiota reduction did not measurably affect α-gal-associated antigen levels under the conditions tested. These preliminary findings suggest that endogenous glycosylation-dependent processes primarily contribute to α-gal antigen biogenesis in *A. americanum* salivary glands.

## 1. Introduction

Alpha-gal syndrome (AGS) is an IgE-mediated food allergy characterized by delayed-type hypersensitivity to galactose-α-1,3-galactose (α-gal), a carbohydrate epitope found on glycoproteins and glycolipids in most non-primate mammals but absent in humans and Old-World primates [1,2,3,4,5]. This absence stems from the evolutionary loss of the α1,3-galactosyltransferase (α1,3GT) gene, leaving humans susceptible to sensitization after exposure to α-gal [1,2,5]. Because α-gal is not produced endogenously, it is recognized as foreign and can stimulate the production of natural anti-α-gal antibodies, primarily IgM and IgG [3,6,7]. In some individuals, repeated exposure promotes class switching to α-gal-specific IgE and leads to delayed allergic reactions 2–6 h after consumption of mammalian meat or other α-gal-containing products [3,4,8]. Tick bites are a major route of sensitization, placing tick saliva at the center of AGS pathogenesis [5,9,10,11]. In the United States, this association is most strongly linked to the Lone Star tick, *A. americanum*, although other tick species have also been implicated worldwide [5,9,12,13,14].

During blood feeding, ticks secrete a complex mixture of salivary proteins, lipids, and glycan-associated molecules that modulate host immunity, facilitate hematophagy, and support pathogen transmission [5,15,16,17,18]. Glycomics and proteomics studies have identified α-gal-containing N-glycans in the salivary glands and saliva of *A. americanum* and *Ixodes scapularis*, whereas these epitopes have not been detected in *Amblyomma maculatum* [10,18]. Repeated tick feeding or exposure to tick salivary gland extracts can also induce anti-α-gal IgG and IgE responses and AGS-like phenotypes in α-gal-deficient mouse models, supporting a role for tick-derived glycoantigens in allergic sensitization [17,19]. In addition, prior work from our laboratory demonstrated that RNAi-mediated knockdown of the α-D-galactosidase (ADGal) enzyme markedly decreased α-Gal abundance and reduced total bacterial load in *A. americanum* salivary glands, whereas silencing β1,4-galactosyltransferase had little effect on α-Gal levels [20]. Previous characterization of the *A. americanum* microbiome in our laboratory showed that *Francisellaceae* predominates in both laboratory-reared and field-collected ticks, although field-collected ticks exhibit greater bacterial diversity and may, in some populations, be dominated by *Coxiellaceae* taxa [21,22]. Therefore, experimentally maintained ticks are expected to harbor a simplified bacterial community largely composed of core taxa [21]. Despite these advances, the mechanisms governing α-gal expression in ticks remain elusive. Not all blood-fed ticks show detectable α-gal, and not every α-gal-positive tick bite leads to AGS. This suggests that α-gal abundance alone does not explain differences in disease risk. In addition to tick-intrinsic factors, such as glycosylation pathways, the microbiome may contribute to α-gal formation or accumulation during feeding, and host-related factors may further shape sensitization at the tick–host interface [5,10,17,19,20,21,23]. Consistent with this view, *Anaplasma phagocytophilum* infection of *I. scapularis* has been shown to increase α-Gal levels and modify the expression of tick galactosyltransferases implicated in α-Gal biosynthesis [24]. More broadly, tick-borne pathogens can remodel tick metabolic and glycosylation-associated pathways, highlighting the potential for microbial signals to influence glycan regulation during tick–pathogen interactions [25].

Among hard ticks in the United States, *A. americanum* and *I. scapularis* show high levels of α-gal in their salivary glands, whereas *A. maculatum* lacks detectable α-gal despite feeding on similar vertebrate hosts [18]. Yet *A. americanum* is strongly linked to AGS, while *I. scapularis* is less consistently associated with clinical sensitization, indicating that α-gal abundance alone does not fully explain disease outcome [5]. These observations also suggest that α-gal expression during blood feeding may depend on both tick microbiota and intrinsic glycosylation or glycan remodeling and salvage pathways. To investigate this possibility, we examined how core microbiome disruption and glycosylation inhibition affect α-gal antigen expression in *A. americanum* salivary glands. Microbiome perturbation was planned to be achieved with tetracycline-induced microbial depletion, and glycan biosynthesis was disrupted using tunicamycin to inhibit N-linked glycosylation and 2-deoxy-2-fluoro-D-galactose (dGalF) to inhibit galactose incorporation. This approach was designed to determine the relative contributions of the core microbiome and tick glycosylation pathways to α-Gal antigen biogenesis during blood feeding, and to identify mechanisms potentially involved in AGS sensitization at the tick–host interface.

## 2. Materials and Methods

### 2.1. Ethics Statement

All animal procedures complied with the National Institutes of Health Guide for the Care and Use of Laboratory Animals and were approved by the Institutional Animal Care and Use Committee at the University of Southern Mississippi (protocol #15101501.8, approved on 23 July 2025; 19041801.3, approved on 15 June 2025).

### 2.2. Ticks, Mice, and Other Animals

Unfed adult Lone Star ticks (*A. americanum*) were obtained from the Oklahoma State University tick rearing facility and maintained in our laboratory at the University of Southern Mississippi as described by Patrick and Hair [26] at room temperature, ~90% relative humidity, and a 14 h light/10 h dark photoperiod before infestation on sheep. Depending on the experimental objective, adult ticks were fed on sheep for 1–11 days before collection. For use in bacterial-depletion experiments, adult ticks were collected at 9–10 days post-infestation once they had repleted. For experiments evaluating α-gal abundance or microbial load in partially fed adults, treated ticks were fed on sheep and collected after 5 days of feeding. For microbial-depletion experiments involving immature ticks, larvae and nymphs were fed on mice under sterile conditions. After tick placement and attachment, mice were housed individually in cages fitted with elevated wire platforms positioned above sterile water collection reservoirs. Fully engorged ticks were recovered following detachment, immediately surface-cleaned, and either processed for downstream analyses or maintained under sterile conditions to molt to the subsequent developmental stage. Alpha-1,3-galactosyltransferase gene knockout (AGTKO) mice on a C57BL/6 background were obtained from Dr. Scott Commins (University of North Carolina at Chapel Hill, Chapel Hill, NC, USA) and bred and maintained in pathogen-free rooms under protocols approved by the University of Southern Mississippi IACUC.

### 2.3. Microbial Depletion in Ticks

Ticks were subjected to a staged tetracycline treatment regimen spanning the life cycle. Adult female ticks were microinjected into the lower right coxa of the ventral side using a Hamilton gastight syringe fitted with a 33-gauge custom 0.5-inch needle with a 45° bevel, delivering tetracycline (1 μL per 100 mg body weight; 10 mg/mL) or sterile water as a control. Injected females were maintained until oviposition [27]. After hatching, larvae *(n* = ~200 per mice) were fed on mice (*n* = 5 for each antibiotic-treated and non-treated group) receiving tetracycline (1.5 g/L) through sterile drinking water, daily oral gavage (200 μL), and intraperitoneal injection (100 μL). Antibiotic treatment began 7 days before infestation and continued throughout larval feeding; control mice (*n* = 5) received sterile water on the same schedule. After molting, nymphs (*n* = 16) were fed on antibiotic-treated mice using the same regimen. A subset of engorged nymphs was allowed to molt into adults. For microbial-depletion experiments, newly molted adult ticks were injected with 1 µL per tick of sterile water (control), tetracycline (3 µg/µL), tunicamycin (0.25 mM/µL), or D-GalF (1 mM/µL), according to the assigned treatment group. Following injection, ticks were allowed to recover for 24 h before being fed on sheep and collected after 5 days of feeding, at the partially engorged stage.

### 2.4. Tick Tissue Dissection and Salivary Gland Extraction

Female ticks partially fed on sheep were dissected, and salivary glands were collected and washed in ice-cold M199 buffer (Gibco, Thermo Fisher Scientific, Waltham, MA, USA). Salivary glands from the same time point (5-day partial feed) were pooled and stored in RNAlater (Life Technologies, Carlsbad, CA, USA) at −80 °C until use, as described elsewhere [16]. Salivary proteins were extracted from five partially blood-fed *A. americanum* ticks per experimental group as previously described [18]. Protein extracts were stored at −80 °C until Western blot analysis.

### 2.5. DNA Extraction and Total Bacterial Load Quantification

Tick eggs, larvae, nymphs, adults, and dissected tissues were collected for analysis. Eggs, larvae, nymphs, and adults were surface sterilized in 2% Sodium hypochlorite (NaOCl), followed by two 5 min washes in 70% ethanol and three rinses in sterile deionized water before DNA extraction. Samples were pooled by stage, with approximately 100 eggs, 10 fed larvae, or 3 fed nymphs per pool. For bacterial load analysis in partially fed adult females, DNA was extracted from dissected salivary glands. Blood-fed female ticks were dissected within 4 h of removal from sheep, and tissues were collected following standard procedures [28]. DNA was extracted using the DNeasy Blood and Tissue Kit (QIAGEN, Germantown, MD, USA) with appropriate controls, and total DNA was quantified with a NanoDrop spectrophotometer (Thermo Fisher Scientific, Waltham, MA, USA). All extraction procedures were performed in a biosafety cabinet to minimize environmental contamination.

Total bacterial load was determined using methods and primers described elsewhere [29]. Briefly, each 25 µL qPCR reaction contained 25 ng tissue DNA, 200 nM 16S rRNA gene primers (Forward: AGAGTTTGATCCTGGCTCAG; Reverse: ATGCTGCCTCCCGTAGGAGT) targeting V1–V2 regions, and iTaq Universal SYBR Green Supermix (Bio-Rad, Hercules, CA, USA) [20]. Cycling conditions were 94 °C for 5 min, followed by 35 cycles of 94 °C for 30 s, 60 °C for 30 s, and 72 °C for 30 s. Each sample was run in technical triplicate, and mean Ct values were used for analysis. Relative bacterial abundance was calculated by normalizing 16S rRNA gene signal to *A*. *americanum* Actin and expressing values relative to the control group. Statistical significance was calculated using an unpaired two-tailed Student’s *t*-test in GraphPad Prism V9.5.1. (GraphPad Software, Boston, MA, USA).

### 2.6. SDS-PAGE and Immunoblotting

SDS-polyacrylamide gel electrophoresis and immunoblotting were performed as described elsewhere [18]. Salivary gland proteins (15 μg) were separated by SDS-PAGE on Mini-PROTEAN TGX Any kD 4–20% gels (Bio-Rad) and transferred to nitrocellulose membranes using a Trans-Blot cell (Bio-Rad) with transfer buffer containing 25 mM Tris-HCl and 192 mM glycine in 20% methanol. Membranes were blocked with 5% Bovine Serum Albumin (BSA; Sigma-Aldrich) in TBS (20 mM Tris-HCl, 150 mM NaCl, pH 7.5) containing 0.1% Tween-20 to reduce nonspecific binding, then incubated with anti-α-galactose (M86) monoclonal IgM antibody (Enzo Life Sciences, Farmingdale, NY, USA) at a 1:10 dilution using an iBind Western device (Life Technologies, Carlsbad, CA, USA). Antigen–antibody complexes were detected with goat anti-mouse IgM secondary antibody conjugated to horseradish peroxidase (Sigma-Aldrich) at a 1:10,000 dilution. Signals were visualized using SuperSignal chemiluminescent substrate (Pierce Biotechnology, Rockford, IL, USA) and a Bio-Rad ChemiDoc XRS system.

### 2.7. Immunolocalization of α-Galactosyl Epitopes

Immunolocalization of α-gal was performed on partially fed salivary glands from *Am. americanum*. Salivary glands were fixed in 1× phosphate-buffered saline (PBS, 137 mM NaCl, 2.7 mM KCl, 10 mM Na_2_HPO_4_, and 1.8 mM KH_2_PO_4_; pH 7.4) containing 4% formaldehyde and stored at 4 °C. Samples were permeabilized with 0.1% Triton X-100 (Sigma-Aldrich, St. Louis, MO, USA) for 30 min, then blocked in 1× PBST (1× PBS containing 0.1% Tween-20) with 5% bovine serum albumin (BSA) for 1 h at room temperature. Tissues were incubated overnight at 4 °C with anti-α-gal IgM antibody (1:20; Enzo Life Sciences, Farmingdale, NY, USA) in 1× PBST containing 5% BSA, followed by incubation with Alexa Fluor 546 goat anti-mouse IgM secondary antibody (1:100; Life Technologies, Carlsbad, CA, USA) and phalloidin-Alexa Fluor 633 (1:100; Abnova, Walnut, CA, USA) in 1× PBS containing 5% BSA for 1 h in the dark. Unless otherwise stated, all incubations and washes were performed on a rocking platform at room temperature. Samples were mounted using ProLong Gold antifade reagent with DAPI (4′,6-diamidino-2-phenylindole; Life Technologies, CA, USA) and imaged on a Leica STELLARIS STED confocal microscope (Leica Microsystems, Wetzlar, Germany) equipped with 40×, 63×, and 100× objectives. For adult ticks, salivary glands from three partially fed individuals were pooled for each biological preparation, and each pooled preparation was processed and imaged once. For nymphs, salivary glands from three individuals were pooled for each biological preparation, and each pooled preparation was processed and imaged separately. Images were acquired using a Leica imaging system and processed using the built-in deconvolution and Lightning modules. Images were exported as acquired and compiled in PowerPoint.

## 3. Results

### 3.1. Microbial Depletion in Ticks via In Vivo and Direct Antibiotic Treatment in Ticks

**Figure 1:**
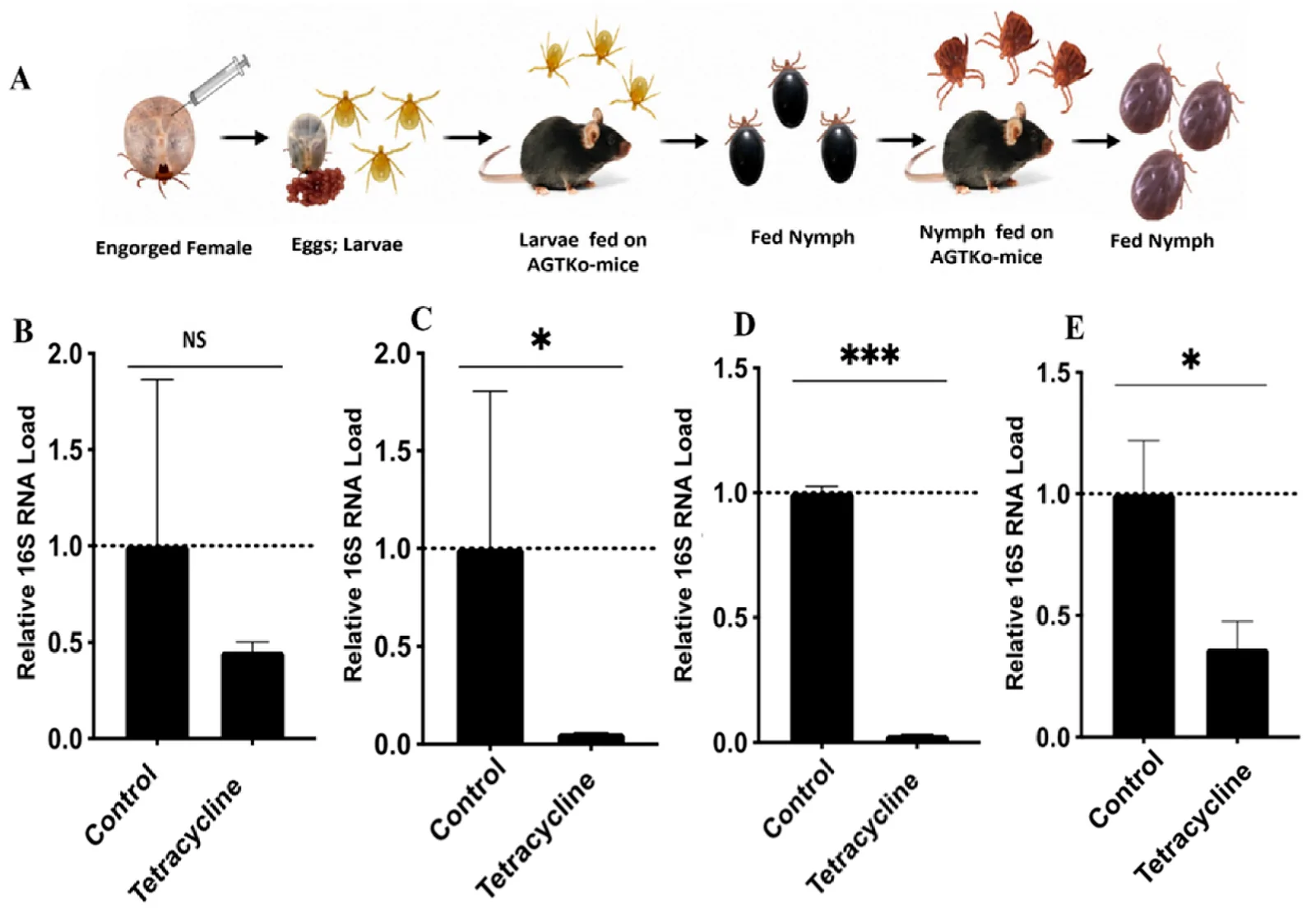
Tetracycline-induced microbial depletion across developmental stages of *A*. *americanum*. (**A**) Schematic overview of the experimental design used to induce microbial depletion across tick developmental stages. Engorged adult female ticks were directly injected with tetracycline. Their progeny were subsequently fed on tetracycline-treated mice during larval and nymphal stages to maintain antibiotic exposure. (**B**–**E**) Relative microbial load in tick developmental stages and adult salivary glands. Relative microbial load was quantified by qPCR targeting the bacterial 16S rRNA gene in (**B**) eggs (100 eggs per pool), (**C**) larvae (15 larvae per pool), (**D**) engorged nymphs (3 nymphs per pool), and (**E**) salivary glands from partially fed adult female ticks from tetracycline-treated and sterile-water-treated groups. DNA was extracted from three independent pools of eggs, larvae, and engorged nymphs and from salivary glands of three individual adult ticks. Relative bacterial load was determined by 16S rRNA qPCR using β-actin as the reference gene, compared between control and treatment groups, and expressed as mean ± SEM. Statistical significance was assessed using an unpaired, two-tailed Student’s *t*-test. NS, not significant (*p* ≥ 0.05); * (*p* < 0.05), significant; *** (*p* < 0.001), significant.

Following continuous tetracycline treatment across tick developmental stages (Figure 1A), total bacterial load in ticks was evaluated by 16S rRNA gene qPCR using DNA extracted from pooled tick samples. Egg pools derived from tetracycline-treated females exhibited variable reductions in microbial load; however, these differences were not statistically significant compared with controls (Figure 1B). In contrast, fed larval pools from the tetracycline-treated group showed a significant reduction in total bacterial load relative to control larvae (Figure 1C). Similarly, bacterial load was significantly reduced in engorged nymph pools from tetracycline-treated ticks compared with control nymphs (Figure 1D).

Salivary glands from partially fed adult female ticks in the tetracycline-treated group also showed reduced relative bacterial load compared with those from sterile-water-treated controls (Figure 1E). Collectively, these data indicate that the tetracycline treatment regimen produced stage-dependent microbial depletion, with no statistically significant reduction detected in eggs but significant reductions observed in larvae, engorged nymphs, and adult female salivary glands. Tetracycline-treated adult ticks tolerated injections without observable adverse effects on behavior or feeding.

### 3.2. Immunolocalization of α-Gal in Salivary Gland Tissues

**Figure 2:**
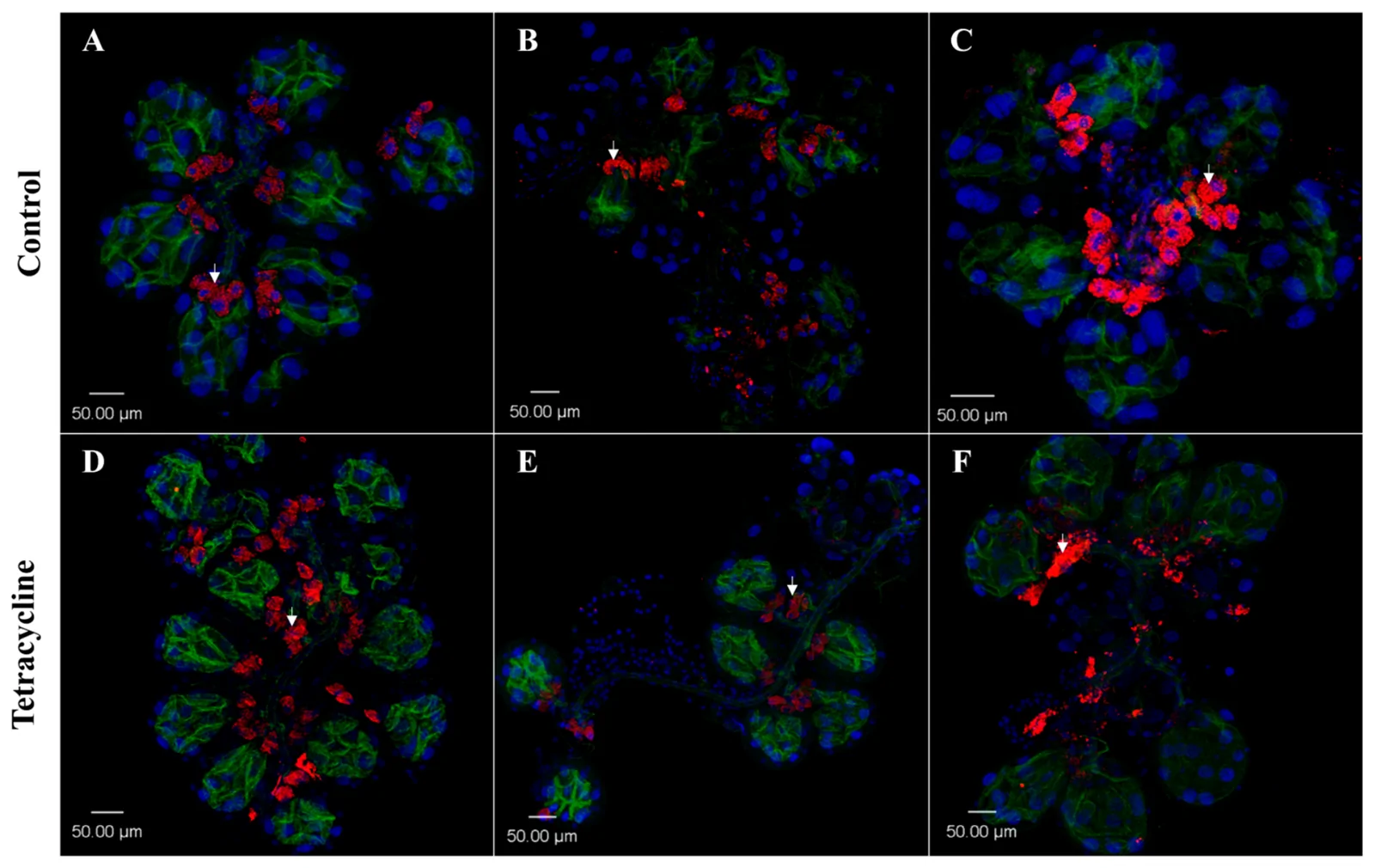
α-Gal immunolocalization in salivary glands of partially fed *A. americanum* nymphs following tetracycline treatment. Representative immunofluorescence images showing α-Gal immunoreactivity in salivary glands of post-feeding nymphs fed on sterile water-treated (**A**–**C**) control mice and (**D**–**F**) tetracycline-treated mice. Salivary glands from three nymphs were pooled for each biological preparation, and each pooled preparation was processed and imaged separately. α-Gal immunoreactivity is shown in red, F-actin stained with phalloidin in green, nuclei stained with DAPI in blue, and merged images are shown in the right panel.

Immunolocalization of α-gal-containing antigens was examined in the salivary glands of partially blood-fed *A. americanum* nymphs and adult females. In control nymphs, α-gal immunoreactivity was detected within the salivary gland acini (Figure 2A–C). α-gal immunoreactivity was also detected in the salivary glands of nymphs exposed to tetracycline through an antibiotic-treated mouse blood meal (Figure 2D–F). The signal was present within the acini, although its distribution varied among individual glands (Supplementary Figures S1 and S2).

**Figure 3:**
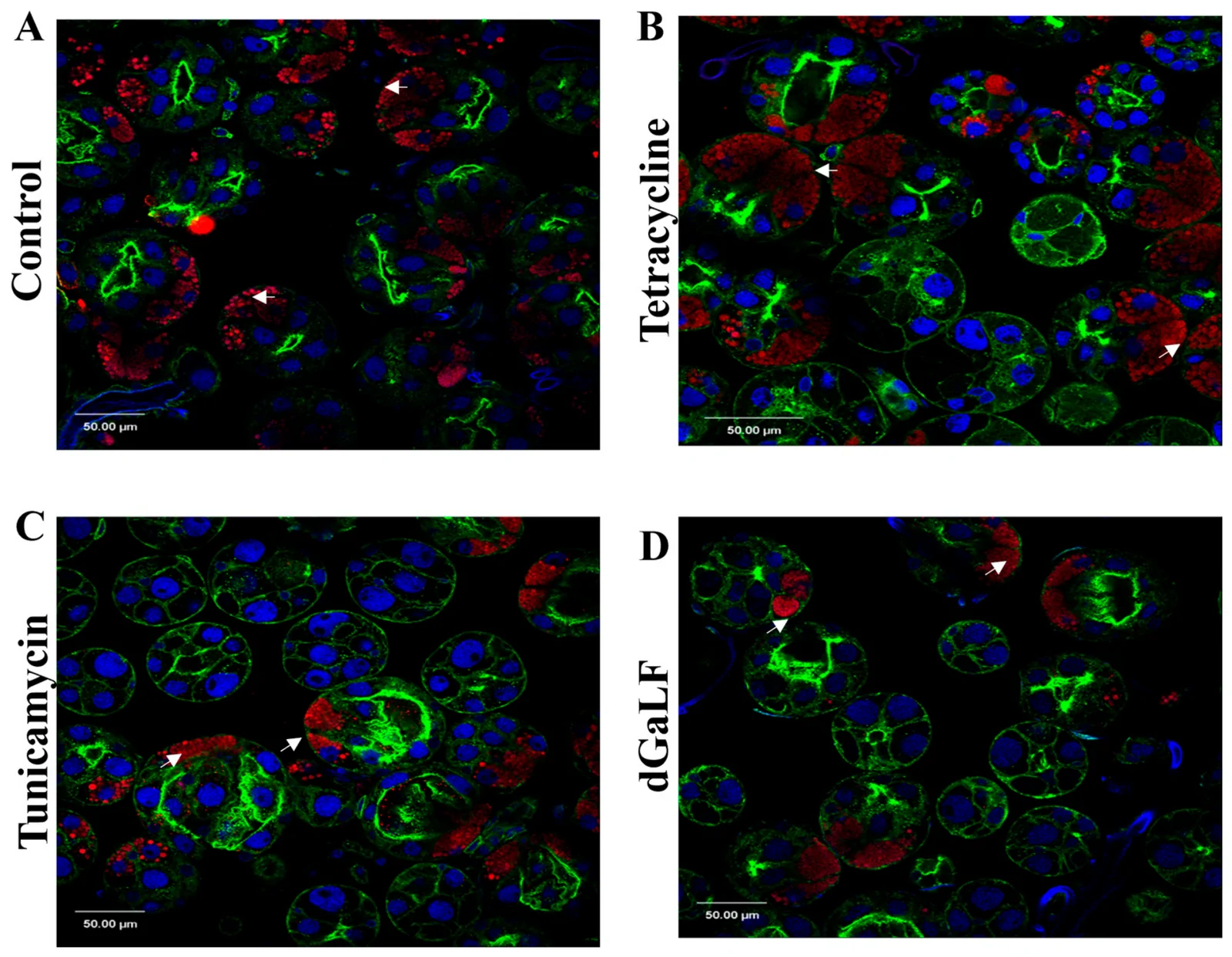
α-Gal immunoreactivity in salivary glands of partially fed adult female *A. americanum* ticks following tetracycline, tunicamycin, or dGalF treatment. Representative immunolocalization images of α-Gal in salivary glands from partially fed adult ticks injected with sterile water as a (**A**) control, (**B**) tetracycline, (**C**) tunicamycin, and (**D**) dGalF. For each biological preparation, salivary glands from three partially fed adult ticks were pooled, processed, and imaged once. α-Gal immunoreactivity is shown in red, phalloidin-stained F-actin in green, and nuclei stained with DAPI in blue.

In adult females, α-gal immunoreactivity was detected within the salivary gland acini in the control, tetracycline-, tunicamycin-, and dGalF-treated groups (Figure 3A–D). α-gal-associated fluorescence was observed in control glands (Figure 3A) and remained detectable following tetracycline (Figure 3B), tunicamycin (Figure 3C), and dGalF treatment (Figure 3D). Signal distribution varied among glands and treatment groups (Supplementary Figure S3). Because fluorescence intensity was not quantitatively assessed, the representative images were used only to document the presence and general localization of α-gal-containing antigens.

### 3.3. Inhibition of N-Glycan Machinery Reduces α-Gal Antigen Abundance in Ticks’ Salivary Glands

**Figure 4:**
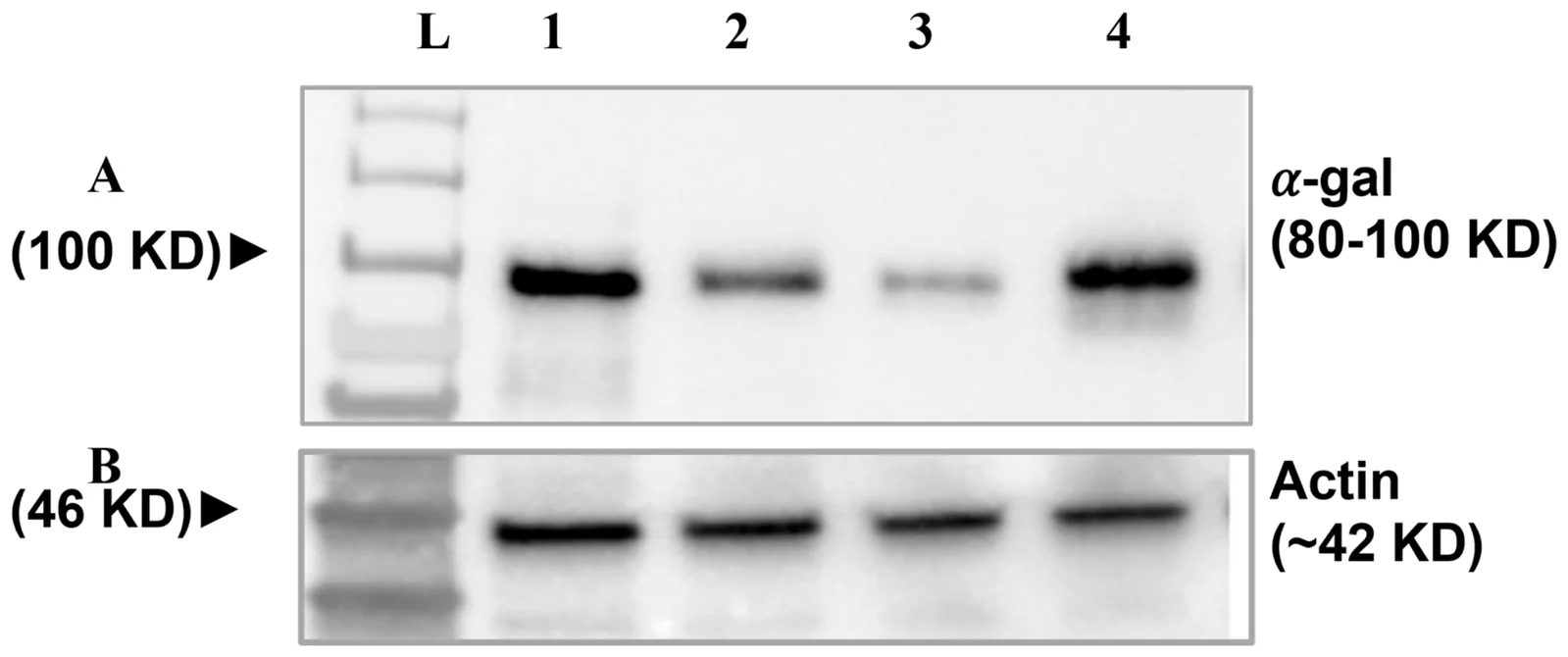
Tick N-glycan machinery is essential for α-Gal-antigen expression in *A. americanum* salivary glands. Western blot showing α-Gal-reactive protein bands at approximately 80–100 kDa in salivary gland (SG) extracts from partially fed female *A. americanum* ticks. α-Gal immunoreactivity was reduced in dGalF- and tunicamycin-treated ticks compared with the sterile-water-treated control, whereas tetracycline-treated ticks showed immunoreactivity comparable to that of controls. L, molecular-weight ladder (NEB, UK); lane 1, sterile-water-treated control; lane 2, ticks treated with the galactosylation inhibitor 2-deoxy-2-fluoro-D-galactose (dGalF); lane 3, ticks treated with the N-glycosylation inhibitor tunicamycin; and lane 4, tetracycline-treated ticks. β-Actin (~42 kDa) was used as a loading control. Salivary glands from five adult female ticks were pooled for each experimental group.

Tick-intrinsic N-glycosylation and galactosyltransferase activity were examined as potential contributors to α-Gal antigen formation in salivary glands during feeding. To target these pathways, ticks were treated with tunicamycin to inhibit N-glycan synthesis or with 2-deoxy-2-fluoro-D-galactose (dGalF) to interfere with galactose incorporation. Qualitative Western blot analysis of salivary-gland protein extracts showed weaker α-Gal-reactive bands in tunicamycin- and dGalF-treated ticks than in sterile-water-treated controls (Figure 4). In contrast, α-Gal immunoreactivity in salivary-gland extracts from tetracycline-treated ticks appeared comparable to that of controls (Figure 4, lanes 1 and 4). For each treatment group, salivary glands from five adult female ticks were pooled for protein extraction and Western blot analysis (Supplementary Figure S4).

## 4. Discussion and Conclusions

The molecular basis of tick-derived galactose-α-1,3-galactose (α-Gal) remains one of the central unanswered questions in α-gal syndrome. Although α-Gal has been detected in the salivary glands and saliva of *A. americanum* and *I. scapularis*, the pathway by which it is generated in ticks remains unclear, and the species-specific differences in α-Gal abundance are still unexplained [5,10,18]. This question is biologically important because α-Gal expression has been linked to tick bites and host sensitization, yet not all α-Gal-positive ticks appear equally capable of inducing the sensitization that leads to alpha-gal syndrome. Defining whether α-Gal arises from intrinsic glycosylation pathways, microbial contributions, or both is therefore central to understanding the biology of α-gal syndrome.

Prior work from our laboratory showed that RNAi-mediated silencing of β1,4-galactosyltransferase did not significantly reduce α-Gal expression in *A. americanum* salivary glands, whereas knockdown of α-D-galactosidase (ADGal) markedly decreased α-Gal abundance as well as total bacterial load in tick salivary glands [20]. These findings prompted us to ask whether α-Gal expression depends not only on specific tick enzymes but also on broader glycan-processing pathways. We therefore tested whether disruption of N-linked glycosylation and related carbohydrate metabolism would affect α-Gal production, with the goal of clarifying whether α-Gal biosynthesis is linked to intracellular glycan processing and galactose handling during tick feeding. Given the association between ADGal silencing and reduced bacterial load observed previously, we also examined whether reduction in the tick microbiome affected α-Gal abundance during feeding. To address this, we tested an approach using continuous tetracycline exposure via feeding on antibiotic-treated mice to induce stage-dependent microbiome depletion across the tick life cycle. Although egg pools from treated females showed only variable and non-significant changes in bacterial load, fed larvae and nymphs showed significant reductions in bacterial abundance, with the greatest depletion observed in partially fed nymphs. This confirmed that the tetracycline regimen produced sustained microbial depletion while preserving tick development. The greater variability observed in egg pools may reflect uneven maternal transmission of symbionts and variable susceptibility of vertically inherited bacteria to antibiotic treatment at the egg stage [30]. Because microbiome composition was not characterized in the present study, the specific bacterial taxa affected by tetracycline treatment remain unknown. We next examined whether tetracycline-mediated microbial depletion altered α-Gal immunoreactivity in tick salivary glands using confocal microscopy. Immunolocalization showed detectable α-Gal immunoreactivity within salivary-gland acini of partially blood-fed *A. americanum* nymphs and adult females. In tetracycline-exposed nymphs, α-Gal staining remained detectable, although its pattern differed among representative glands relative to controls. Similarly, adult salivary glands from tetracycline-treated ticks retained α-Gal-positive staining. These observations indicate that tetracycline treatment did not abolish detectable α-Gal immunoreactivity in salivary-gland acini. Quantitative comparison of fluorescence intensity among treatment groups was not performed because partially fed adult salivary glands contain heterogeneous acini and cell types with variable staining patterns and secretory states; consequently, fluorescence intensity in individual confocal fields may not accurately represent total α-Gal abundance within the entire salivary gland. The confocal images were therefore interpreted qualitatively to document the presence and general distribution of α-Gal immunoreactivity. Although the specificity of the M86 antibody for α-Gal has been previously validated [18], the absence of an appropriate negative staining control limits our ability to distinguish α-Gal-specific labeling from background or nonspecific fluorescence in this analysis. Moreover, reduced total bacterial load does not exclude contributions from residual bacteria, individual bacterial taxa, or indirect antibiotic-associated effects on tick physiology.

To examine the contribution of the N-glycan biosynthesis pathway to α-Gal expression in ticks, we used tunicamycin to block the initiation of N-linked glycosylation, a well-established inhibitor of the first step in the dolichol-linked pathway, and 2-deoxy-2-fluoro-D-galactose (dGalF) to interfere with galactose incorporation and glycoprotein glycosylation [31,32,33]. In the qualitative Western blot analysis, α-Gal-reactive bands were weaker in salivary-gland protein extracts from tunicamycin and dGalF-treated ticks than in sterile-water-treated controls. In contrast, α-Gal immunoreactivity in salivary-gland extracts from tetracycline-treated ticks appeared comparable to that of controls. Tunicamycin has been widely used to assess the contribution of dolichol-linked N-glycan precursor synthesis to glycoprotein folding, maturation, trafficking, and surface expression [34,35]. By blocking the transfer of GlcNAc-1-phosphate to dolichol phosphate, tunicamycin prevents formation of the lipid-linked oligosaccharide precursor required for N-glycosylation [35]. In the present study, the reduction in α-Gal-associated antigen signal following tunicamycin and dGalF treatment suggests that endogenous glycosylation-dependent processes contribute to α-Gal antigen biogenesis in tick salivary glands. The lack of a detectable effect following microbial reduction further indicates that the measured α-Gal-associated antigen signal is not solely dependent on tetracycline-sensitive tick-associated microbiota. However, these pharmacologic treatments do not establish the precise effects on α-Gal epitope abundance, structure, carrier molecules, or the N-glycan profile. In addition, because salivary glands from five adult female ticks were pooled per treatment condition and additional independent biological pools were not available for densitometric quantification or statistical testing, the Western blot observations should be interpreted as qualitative, preliminary evidence.

We were unable to confirm α-Gal abundance in nymph salivary glands because of technical limitations in obtaining sufficient material for Western blot analysis. Nevertheless, the Western blot data provide direct evidence that tunicamycin and dGalF reduce N-linked α-Gal antigens in adult salivary glands. Together, these findings indicate that intact N-glycan biosynthesis and galactose incorporation are required for robust α-Gal expression and point to a dominant role for the tick’s intrinsic glycosylation machinery. This interpretation helps distinguish endogenous glycosylation-dependent mechanisms from core-microbiome-dependent effects in *A. americanum*. Although tetracycline treatment produced measurable bacterial depletion and did not show an impact on α-Gal abundance, the substantially stronger response to tunicamycin and dGalF indicates that endogenous glycan biosynthesis and maturation possibly are principal determinants of α-Gal expression in *A. americanum* salivary glands.

This interpretation is also consistent with our earlier RNAi data showing that ADGal contributes to α-Gal abundance and suggesting that glycan turnover, recycling, and intracellular processing influence the pool of substrates available for α-Gal formation in tick salivary glands [20]. The dependence of α-Gal expression on glycan biosynthesis is particularly notable because no canonical α-1,3-galactosyltransferase has been identified in currently available *A. americanum* genomic or transcriptomic resources. These findings therefore suggest that α-Gal formation may depend on an uncharacterized enzymatic activity or a tick-specific glycosylation pathway capable of generating α-Gal-bearing glycans through N-glycan remodeling, salvage, or synthesis during glycan maturation. This possibility is consistent with glycomic and glycoproteomic studies showing that *A. americanum* and *I. scapularis* express complex bi- and tri-antennary N-glycans with terminal Gal-Gal and α-Gal epitopes, together with a limited set of α-Gal-containing glycolipids in salivary secretions [10,18,20]. At the same time, genomic and transcriptomic surveys have not identified canonical GT6 α-1,3-galactosyltransferases in *Ixodida* but have revealed expansion of GT7, GT31, and GT32 galactosyltransferase families in ticks [5,24]. Collectively, these observations and our inhibition data support the hypothesis that α-Gal in tick salivary glands arises through a noncanonical, tick-specific N-glycan biosynthetic pathway and associated glycan processing or remodeling. Within this expanded repertoire, A4GALT-like GT32 enzymes are attractive candidates for alternative α-Gal synthases because of their conserved anomeric specificity and lineage-specific duplication in ticks. However, identifying the responsible enzymatic activity will require targeted biochemical and genetic studies, as well as detailed genome annotation and mapping. These results also help explain why α-Gal abundance varies across tick species and feeding stages. Previous studies showed that α-Gal is detectable in *A. americanum* and *I. scapularis* but absent in species such as *A. maculatum* and *Dermacentor variabilis*, and that α-Gal abundance increases during blood feeding, particularly in partially fed ticks [18,20].

How α-Gal reaches and is exposed to the host remains unclear. One possibility is that it is carried on salivary glycoproteins that pass through the classical ER-Golgi secretory pathway and are released during blood feeding. Another possibility is that α-Gal-containing glycoconjugates are packaged into extracellular vesicles (EVs). Tick saliva contains EVs that carry bioactive proteins and other molecules into host tissues, where they can modulate immune responses and support blood feeding [36,37]. However, α-Gal has not yet been shown within tick-derived EVs. Thus, our findings are consistent with either direct secretion of α-Gal-bearing salivary glycoconjugates or EV-mediated delivery, and they point to the need for future glycomic and glycoproteomic studies to identify the molecules responsible for α-Gal transfer at the tick–host interface. This study was limited to salivary gland tissues and protein extracts, so we did not identify the specific α-Gal-bearing molecules responsible for host exposure. In addition, tetracycline treatment broadly altered the microbiome and may have affected tick physiology beyond bacterial depletion, particularly in nymphs. Future studies using glycomic, glycoproteomic, and lipidomic approaches should define the molecular carriers of α-Gal, while targeted microbiome or genetic manipulations should be used to identify the pathways that regulate its production. In addition to microbiome composition and glycosylation flux, other intrinsic factors such as feeding stage-dependent salivary gland activation, regulation and differential expression of tick galactosyltransferases and glycosidases among ticks, and the availability of host-derived glycan substrates are also likely to modulate α-Gal expression and will need to be considered together with the intrinsic N-glycan biosynthetic machinery in future studies.

Previous studies from our laboratory characterized the core microbiome of laboratory-reared and field-collected *A. americanum* ticks, identifying *Francisellaceae* as the dominant bacterial family and greater microbial diversity in field-collected ticks [21]. In experimentally maintained, laboratory-reared ticks, the microbiome is expected to consist predominantly of the core bacterial community described above. In contrast, under natural conditions, tick-borne pathogens such as *Ehrlichia* spp., *Anaplasma phagocytophilum*, and *Borrelia* spp. may alter salivary gene expression and increase α-Gal signatures, suggesting that infection could further influence α-Gal antigen production and glycosylation or glycan remodeling processes during feeding.

Microbiome composition, basophil activation test as a measure of α-Gal antigenic potential, and α-Gal N-glycan structures were not directly assessed in this study. Future work will address these limitations using independent biological replicates, quantitative antigen and N-glycan analyses, functional immune assays, detailed microbiome profiling, and glycomic, glycoproteomic, and lipidomic approaches to identify the molecular carriers of α-Gal and define the pathways regulating its production. Overall, this preliminary study demonstrates an approach to deplete microbial load and α-Gal biogenesis in ticks. Preliminary findings from this study indicate that α-Gal biogenesis in *A. americanum* salivary glands is linked to intrinsic N-glycan biosynthetic processes and may also be influenced by a more limited, stage-specific contribution from the core microbiome. By linking α-Gal abundance with glycosylation activity, feeding stage, and salivary-gland localization, these findings provide a framework for mechanistic studies of α-Gal synthesis and secretion in ticks and for investigating their potential contribution to host sensitization in α-gal syndrome.

## Data Availability

The original contributions presented in this study are included in the article/Supplementary Materials. Further inquiries can be directed to the corresponding author.

## Supplementary Materials

The following supporting information can be downloaded at: https://www.mdpi.com/article/10.3390/microorganisms14102375/s1, Figure S1: α-gal immunolocalization in nymphs–salivary glands; Figure S2: α-gal immunolocalization in nymphs–salivary glands- Z-stacking videos; Figure S3: α-gal immunolocalization in nymphs–salivary glands; Figure S4: Western blot images.
